# Impact of a Timely Diagnosis of Early Alzheimer’s Disease on Functional Outcomes

**DOI:** 10.1002/alz.092674

**Published:** 2025-01-09

**Authors:** Becky A Briesacher, Ning A Zhang, Timothy R Juday, Feride H Frech, Richard Batrla

**Affiliations:** ^1^ Northeastern University, Boston, MA USA; ^2^ Eisai Inc., Nutley, NJ USA

## Abstract

**Background:**

The National Plan to Address Alzheimer’s Disease (AD) prioritizes timely diagnosis of mild cognitive impairment (MCI) as one of its key goals. Studies describing the downstream consequences of not having a timely diagnosis in this vulnerable population are limited. The study objective will evaluate the relationship between a timely MCI diagnosis and decline in functional outcomes compared to a missed diagnosis.

**Method:**

This observational study utilizes 2008‐2020 data from the Health and Retirement Study (HRS), a longitudinal panel study that surveys a representative sample of 20,000 people age >50 years in the US every 2 years. Our study is research in progress; final data will extend through 2022. Cognition was assessed by a validated tool, Telephone Interview for Cognitive Status (TICS). A timely diagnosis of MCI is defined as a self or proxy report of a healthcare provider diagnosis in the HRS within 2 years of an MCI‐qualifying score on the TICS. Survival analyses will be used to assess longitudinal change every two years between diagnosed and missed diagnosis groups.

**Result:**

Preliminary analyses identified a study population of 4712 patients with an MCI‐qualifying score; 303 (6.4%) reported a timely MCI diagnosis. Ongoing analyses will shed light on the impact of a timely MCI diagnosis on functional outcomes.

**Conclusion:**

Final analyses will clarify results and provide an indication of the impact of a timely MCI diagnosis on functional outcomes.

